# Supplementary material for: Open Science Drone Toolkit: Open source hardware and software for aerial data capture
Source: PLoS One. 2023 Apr 6;18(4):e0284184. doi: 10.1371/journal.pone.0284184 (PMC10079087; doi:10.1371/journal.pone.0284184)
Supplement: S1 File — Complete manuscript in Spanish: “Herramientas de código abierto para la captura de datos aéreos mediante drones”. (PDF) [file pone.0284184.s001.pdf]

Herramientas de código abierto para la captura de datos aéreos mediante drones

Gustavo Pereyra Irujo[1*], Paz Bernaldo[2], Luciano Velázquez[3], Antoni Pérez[4], Celeste Molina Favero[5], Alejandrina Egozcue[6]

[1] Instituto Nacional de Tecnología Agropecuaria, Consejo Nacional de Investigaciones Científicas y Técnicas, Balcarce, Argentina [2] Investigadora independiente, Melipilla, Chile [3] Facultad de Ciencias Agrarias, Universidad Nacional de Mar del Plata, Balcarce, Argentina [4] Investigador independiente, Santiago, Chile [5] Unidad Integrada Balcarce, Universidad Nacional de Mar del Plata, Instituto Nacional de Tecnología Agropecuaria, Balcarce, Argentina [6] Investigadora independiente, Balcarce, Argentina
[*] pereyrairujo.gustavo@conicet.gov.ar

Resumen

A pesar del mayor acceso a publicaciones y datos científicos como resultado de las iniciativas de ciencia abierta, el acceso a las herramientas científicas sigue siendo limitado. Los vehículos aéreos no tripulados (VANT o drones) pueden ser una herramienta poderosa para la investigación en disciplinas como la agricultura y las ciencias ambientales, pero su uso está actualmente dominado por herramientas propietarias y de código cerrado. El objetivo de este trabajo fue recopilar, seleccionar, organizar y evaluar un conjunto de herramientas de código abierto para la captura de datos aéreos con fines de investigación. El "Open Science Drone Toolkit" fue creado a través de un proceso colaborativo e iterativo en el que participaron más de 100 personas en cinco países,e incluye un dron autónomo de hardware abierto, software de código abierto, y guías y protocolos que permiten al usuario realizar todas las tareas necesarias para obtener datos a partir de imágenes aéreas. Los datos obtenidos con este conjunto de herramientas sobre un campo de trigo fueron comparados con datos de imágenes satelitales y un sensor portátil comercial, y se encontró una alta correlación para ambos instrumentos. Estos resultados demuestran la posibilidad de capturar datos aéreos aptos para su uso en investigación utilizando software y hardware de código abierto, asequibles, accesibles y personalizables, y protocolos de trabajo abiertos.

Cita: Pereyra Irujo G, Bernaldo P, Velázquez L, Pérez A, Molina Favero C, Egozcue A. (2022) Herramientas de código abierto para la captura de datos aéreos mediante drones [Open Science Drone Toolkit: open source hardware and software for aerial data capture]. doi: 10.5281/zenodo.7093644

Introducción

La ciencia moderna ha tenido a la apertura como uno de sus pilares desde sus inicios (Hull, 1985), a través de la publicación de teorías y los datos en los que éstas se basan, y del fomento de la replicación, el escrutinio y la duda

(Boulton et al., 2012). En las últimas décadas, las tecnologías de la información han permitido el surgimiento de un movimiento global de "ciencia abierta" que busca no sólo aumentar la transparencia y la difusión de los procesos y productos científicos, sino también permitir una colaboración, participación e inclusión más amplias en la ciencia (Bahlai et al. , 2019; UNESCO, 2021; Wolff y Schlagwein, 2021). Aunque el acceso a publicaciones y datos científicos ha aumentado significativamente en los últimos años (Boulton et al., 2011; Forero et al., 2021; Himmelstein et al., 2018; Piwowar et al., 2018), el acceso a las herramientas necesarias para obtener o analizar datos (es decir, instrumentos científicos, materiales y software) sigue siendo una de las principales barreras para aumentar la participación en la producción científica, y la replicación o la reproducción de resultados publicados (Arancio y Dosemagen, 2022; Walters, 2020).

El software de código abierto son aquellos programas informáticos que se licencian para que el usuario tenga la libertad de copiarlos y redistribuirlos, tener acceso al código fuente y realizarles mejoras, entre otros derechos (Open Source Initiative, 2007). De manera similar, el hardware de código abierto son aquellos objetos u artefactos cuyo diseño está disponible para que cualquier persona pueda estudiarlos, modificarlos, distribuirlos, fabricarlos y venderlos (Open Source Hardware Association, 2016). Se considera que el software científico de código abierto y los instrumentos y materiales científicos abiertos brindan una serie de ventajas sobre las alternativas propietarias: i) al ser gratuitos (en el caso del software) o generalmente más asequibles (en el caso del hardware), permiten a más personas participar en actividades científicas, especialmente investigadores no profesionales o aquellos con un presupuesto limitado; ii) la reproducibilidad de los resultados publicados o los intentos de replicación están menos limitados por la falta de acceso a las herramientas que se usaron originalmente; iii) tener acceso al código del software o al diseño del hardware permite una mejor comprensión del funcionamiento de la herramienta y de los métodos o algoritmos que implementa; y iv) es posible personalizar las herramientas para adaptarlas a nuevos usos o contextos locales (Chagas, 2018; Diederich et al., 2022; Ravindran, 2020; Walters, 2020).

Los vehículos aéreos no tripulados (VANT, generalmente llamados “drones”) pueden ser una poderosa herramienta para la investigación en disciplinas como la agricultura y las ciencias ambientales, permitiendo la captura de imágenes aéreas de alta resolución con gran velocidad y flexibilidad (Chabot, 2018). El uso de drones en investigación está creciendo rápidamente, pero está dominado por herramientas de código cerrado: en una revisión reciente de la literatura sobre aplicaciones en monitoreo agroambiental, más del 80 % de los estudios utilizaron drones de código cerrado y más del 90 % de los estudios utilizó un software comercial de código cerrado para el procesamiento de imágenes (Eskandari et al., 2020). Los paquetes comerciales para la utilización de drones suelen requerir una inversión inicial significativa, suscripciones mensuales de software o conexión a internet para procesamiento en la nube, lo cual puede constituir una barrera para muchos usuarios de bajos recursos (Paneque-Gálvez et al., 2017; Vargas-Ramírez y Paneque-Gálvez, 2019) y, por lo general, funcionan como una "caja negra" que ofrece a los usuarios poca información sobre su funcionamiento interno y una personalización limitada (Pell et al., 2022). Además, cuando estas soluciones se implementan en países en desarrollo, existe el riesgo de que resulten en una capacidad limitada de reparación, dependencia tecnológica y prácticas extractivas (Hanrahan et al., 2021).

Para un uso típico de drones en investigación ambiental o agrícola, el dron debe poder posicionarse de manera confiable y precisa sobre el terreno estudiado y capturar imágenes que luego se puedan procesar para obtener una imagen de alta calidad del área estudiada y extraer datos útiles (Assmann et al., 2019; Rusnák et al., 2018; Tmušić et al., 2020). Aunque ya existen herramientas de hardware y software de código abierto desarrolladas para distintos pasos individuales de este proceso, nuestra pregunta era si era posible realizar la totalidad de estos pasos utilizando herramientas abiertas. El objetivo de este trabajo fue abordar esta pregunta mediante la recopilación, selección,

organización y evaluación de un conjunto integral de herramientas de código abierto para la captura de datos aéreos con fines de investigación. El resultado de estas acciones es el Open Science Drone Toolkit (OSDT), que se presenta en detalle en este trabajo, y está además disponible en línea en https://vuela.cc/toolkit.

Proceso de diseño

El OSDT se desarrolló como parte de “Vuela” (Bernaldo y Pereyra Irujo, 2018; Proyecto Vuela, 2018), un proyecto de investigación-acción que tuvo como objetivo combatir la falta de acceso a la creación de conocimiento científico y tecnológico, explorando una forma alternativa de desarrollar herramientas científicas. El conjunto de herramientas se armó a través de un proceso colaborativo e iterativo, que involucró el trabajo de más de 100 personas entre 2017 y 2019, en más de 30 talleres presenciales, en cinco países (Argentina, Brasil, Chile, Paraguay y Uruguay), y mediante colaboración online permanente. Los miembros de la "tripulación" eran estudiantes secundarios, científicos tradicionales, técnicos, aficionados a la electrónica, periodistas, miembros de comunidades local, desarrolladores de software autodidactas, e incluían tanto académicos como personas sin antecedentes académicos o tecnológicos formales, y personas con y sin experiencia en construir o utilizar drones. Se trabajó también en diferentes idiomas: español, criollo haitiano, francés, portugués e inglés.

El Open Science Drone Toolkit es un conjunto de herramientas de hardware y software, guías y protocolos para permitir al usuario realizar todas las tareas necesarias para obtener datos a partir de imágenes aéreas, como se detalla en la Tabla 1. Estos pasos representan un caso de uso típico, pero se pueden modificar de acuerdo con el tipo de investigación.

Tabla 1. Tareas que se pueden realizar utilizando Open Science Drone Toolkit para obtener datos a partir de imágenes aéreas.

Tarea	Detalle
**0. Armar el conjunto de herramientas**	Construir o adquirir el dron, la cámara y otros componentes de hardware, instalar los componentes de software y realizar las configuraciones necesarias, siguiendo la guía de ensamblaje del kit de herramientas.
**1. Identificar un área de estudio**	Especificar los límites del área que se medirá, ya sea *in situ* o utilizando imágenes aéreas o satelitales georreferenciadas.
**2. Diseñar un plan de vuelo**	Diseñar la ruta de vuelo que seguirá el dron sobre el área de estudio, así como la altitud y la velocidad del vuelo, teniendo en cuenta la resolución de muestreo deseada (cm por píxel), la superposición requerida para la unión de las imágenes y las limitaciones técnicas del dron y cámara
**3. Seleccionar los parámetros de la cámara**	Seleccionar los parámetros óptimos de la cámara (velocidad del obturador, sensibilidad ISO, intervalo de captura automática) de acuerdo con las condiciones atmosféricas en el momento del vuelo
**4. Hacer un vuelo autónomo**	Realizar un vuelo autónomo guiado por satélite sobre el área de estudio, siguiendo el plan de vuelo diseñado, capturando las imágenes necesarias usando parámetros de cámara fijos
**5. Georreferenciar las imágenes capturadas**	Asignar coordenadas a cada imagen de acuerdo con la ubicación precisa registrada por el dron en el momento de la captura
**6. Procesar las imágenes para obtener un mosaico**	Procesar las imágenes capturadas para obtener una imagen ortorrectificada y georreferenciada de la totalidad del área relevada

**7. Analizar el mosaico para obtener datos**	Procesar el mosaico para obtener datos relevantes para la pregunta de investigación, a partir de la imagen completa o para áreas específicas
**8. Organizar y compartir la información**	Organizar y visualizar todos los datos generados (imágenes sin procesar, mosaicos, límites de área, plan de vuelo y registros de telemetría) y metadatos (nombre y descripción del vuelo, fecha y hora, ubicación, etc.) y agrupar todos los archivos para almacenarlos y compartirlos

Uno de los primeros objetivos del proyecto fue poner en práctica una de las libertades comúnmente menos aprovechadas del hardware de código abierto: la libertad de modificar un diseño existente. En lugar de desarrollar un dron desde cero, el proyecto comenzó replicando, probando e identificando mejoras potenciales para un dron de código abierto ya disponible llamado “Flone” (Amorós et al., 2015). El diseño original tenía capacidades limitadas para realizar las tareas requeridas enumeradas en la Tabla 1, por lo que se necesitaron una serie de cambios para usar el dron con fines de investigación: aumentar la autonomía y el área que el dron podía cubrir de manera segura, agregar capacidad de navegación satelital, aumentar la capacidad de carga útil, y mejorar la estabilidad de la cámara. Simultáneamente a este proceso iterativo de desarrollo de hardware se llevó a cabo una cuidadosa selección de herramientas de software de código abierto (y el desarrollo de otras nuevas) para realizar cada una de las tareas enumeradas en la Tabla 1, y el desarrollo de protocolos y guías detalladas. El conjunto de herramientas resultante se describe en la siguiente sección.

Componentes del OSDT

Los componentes del OSDT se enumeran en la Tabla 2. El componente principal es el dron de hardware abierto, desarrollado especialmente para este conjunto de herramientas. Otros componentes de hardware del conjunto de herramientas no son de código abierto, pero son en su mayoría equipos o accesorios estándar que se pueden reemplazar fácilmente. Los componentes de software son todos de código abierto, seleccionados por ser adecuados para cada tarea, o desarrollados especialmente. Finalmente, la documentación incluye guías de construcción y uso del conjunto de herramientas.

Tabla 2. Resumen de los componentes de hardware, software y documentación del Open Science Drone Toolkit.

Tipo de componente	Componente	Tarea en la que se utiliza
**Hardware**	Computadora (PC genérica con sistema operativo Windows)	0 a 8
	Smartphone (teléfono inteligente genérico con sistema operativo Android)	1
	Cámara (cámara fotográfica de bolsillo marca Canon compatible con el software CHDK)	3, 4
	Dron (dron OVLI)	4
	Radiocontrol (radiocontrol genérico de 6 canales)	4
	Baterías y cargador (batería de litio-polímero de 3 celdas y cargador balanceador genéricos)	4

**Software**	Registrador de ubicación (GPS Logger)	1
	Estación de tierra (Mission Planner)	2, 4, 5
	Controlador de cámara (CHDK)	3, 4
	Piloto automático (ArduCopter)	4
	Procesamiento de imágenes (OpenDroneMap)	6
	Procesamiento y análisis de mosaicos (QGIS)	7
	Manejo de datos (Bitácora)	8
**Documentación**	Guía de construcción	0
	Guía de uso	1 a 8

El dron OVLI (acrónimo de “Objeto Volador Libre”) es un cuadricóptero (es decir, un helicóptero de cuatro hélices), equipado con un piloto automático con acelerómetro, giroscopio, barómetro y sensores GNSS (sistema global de navegación por satélite, por su sigla en inglés) que permiten un vuelo totalmente autónomo. Este piloto automático es una placa Pixhawk de código abierto (Meier et al., 2012; Pixhawk Project, 2014), que ejecuta el firmware de código abierto ArduPilot/ArduCopter (ArduPilot Project, 2016; Short et al., 2015). El OVLI tiene un diámetro de su estructura de 395 mm y pesa 0,773 kg sin baterías. El material de la estructura es MDF (Medium Density Fibreboard) cortado con una máquina de corte láser siguiendo un archivo con el plano, que se puede editar fácilmente para modificar la estructura del dron. Se eligió este material porque está ampliamente disponible, es de bajo costo y fácil de ensamblar, reparar y modificar. El diseño final del OVLI se muestra en la Fig. 1, en comparación con el diseño original del “Flone” en el cual está basado.

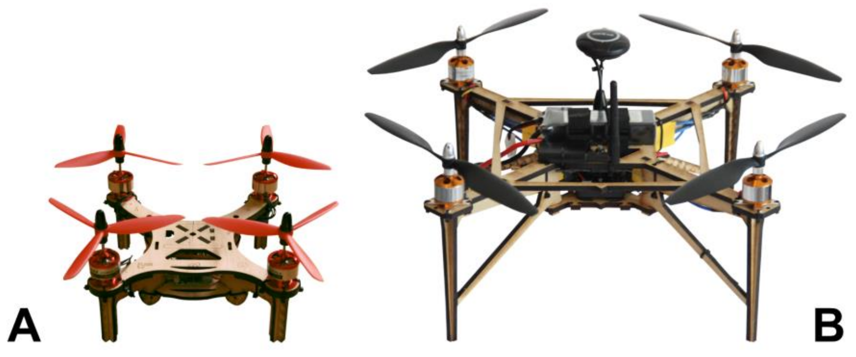

Figura 1. El “Flone” y el “OVLI”. (A) diseño original de “Flone” (Amorós et al., 2015) sobre el cual se desarrollaron las versiones posteriores del dron. (B) El dron “OVLI” que se desarrolló como parte del Open Science Drone Toolkit.

El dron OVLI tiene una capacidad de carga útil de alrededor de 500 g, suficiente para una cámara RGB (rojo-verde-azul, por sus iniciales en inglés, *i.e.*, el espectro visible al ojo humano) de alta resolución, una cámara multiespectral u otros sensores. El tiempo máximo de vuelo es de 11 minutos, usando una batería de 5000 mAh y llevando una cámara de 141 g como carga útil, medido desde el despegue hasta que se activa la alarma de batería baja, quedando aproximadamente un 30% de la capacidad de la batería.

La operación del dron OVLI se puede realizar mediante un radiocontrol manual, pero para utilizarlo con fines de investigación generalmente es conveniente volar el dron de forma autónoma, utilizando un plan de vuelo programado previamente. La planificación generalmente comienza con la identificación del área que se va a relevar (tarea nro. 1 en la Tabla 1), lo que se puede hacer rodeando físicamente el área con un teléfono inteligente y usando la aplicación de código abierto GPSLogger (Tabla 2). El archivo resultante con las coordenadas se carga en el software de código abierto Mission Planner (Tabla 2) para diseñar el plan de vuelo (tarea nro. 2 en la Tabla 1), de acuerdo a la resolución de imagen deseada, y considerando el tiempo máximo de vuelo del dron y demás limitantes. Este plan de vuelo se carga en el dron OVLI, que luego puede volar de forma autónoma mientras captura las imágenes (tarea 4 en la Tabla 1). Durante el vuelo, el software Mission Planner también se usa para ver datos de telemetría en vivo, tales como la posición del dron, el voltaje de la batería, la altitud, la velocidad, etc.

La cámara seleccionada para este conjunto de herramientas es una cámara de bolsillo RGB de 12 megapíxeles (Canon PowerShot ELPH100HS o equivalente). Se seleccionó este tipo de cámara por dos razones principales: 1) las cámaras digitales de bolsillo suelen tener un obturador mecánico, lo que significa que cuando se captura una imagen, todos los píxeles se capturan al mismo tiempo, mientras que otras cámaras, tales como las “cámaras deportivas”, suelen tienen un obturador electrónico de tipo “rolling shutter”, en el que el sensor captura las imágenes línea por línea, lo que podría introducir distorsiones en la imagen (Vautherin et al., 2016); 2) la mayoría de las cámaras Canon tienen la posibilidad de ser “hackeadas” mediante el software de código abierto CHDK (Canon Hack Development Kit) (CHDK Development Team, 2007), que permite configurar la cámara para capturar imágenes automáticamente y configurar manualmente parámetros de la cámara (p. ej., velocidad de obturación y sensibilidad ISO; tarea nro. 3 en la Tabla 1) para capturar imágenes nítidas, bien expuestas y adecuadas para su posterior procesamiento y extracción de datos (O'Connor et al., 2017). Este tipo de cámara no proporciona datos de localización GPS de las imágenes (que son necesarios para obtener posteriormente un mosaico georreferenciado), por lo que esta información hay que recuperarla del registro de vuelo del dron. Este proceso se denomina georreferenciación (tarea nro. 5 en la Tabla 1) y se puede realizar con el software de estación terrestre Mission Planner. Se ha demostrado que este tipo de cámaras RGB de bolsillo son útiles para la medición de índices de vegetación (Svensgaard et al., 2019). Además, estas cámaras pueden ser modificadas reemplazando el filtro estándar de infrarrojo cercano por un filtro apropiado, obteniendo así una cámara capaz de detectar dos bandas en el espectro visible y una banda en el infrarrojo cercano (Sankaran et al., 2018; Jewan et al., 2018).

Luego, las imágenes capturadas deben fusionarse para obtener una imagen rectificada y georreferenciada del área relevada completa, lo que se conoce como ortomosaico (tarea nro. 6 en la Tabla 1). Actualmente, un software de código abierto adecuado para realizar esta tarea es OpenDroneMap (OpenDroneMap Authors, 2020), que ha demostrado proporcionar resultados de alta calidad, comparables a los obtenidos con paquetes comerciales ampliamente utilizados (Pell et al., 2022). El siguiente paso es extraer información de la imagen del ortomosaico, el cual depende en gran medida de la pregunta de investigación que se esté abordando. El software de código abierto QGIS (QGIS Development Team, 2002) puede usarse para abrir el ortomosaico georreferenciado y calcular índices de vegetación, medir áreas y muchas otras tareas de extracción de datos (tarea nro. 7 en la Tabla 1).

La aplicación de código abierto “Bitácora” (https://vuela.cc/bitacora), desarrollada especialmente para el OSDT, ayuda a visualizar y organizar todos los archivos, imágenes y metadatos generados durante todo el proceso, para almacenarlos, compartirlos o continuar su análisis. El usuario sólo necesita guardar en una misma carpeta todos los archivos generados durante un vuelo (polígono del área relevada, plan de vuelo, imágenes capturadas, mosaico, modelo de elevación, etc.), y el programa generará automáticamente un mapa visualizando los archivos y una tabla con información del vuelo (fecha y hora del vuelo, ubicación, altitud, velocidad, nombres de archivos relevantes; Fig. 2). Esta información también se guarda en formatos abiertos compatibles con otros programas (tabla de información de vuelo en formato csv, mapa de vuelo en formato png y kml).

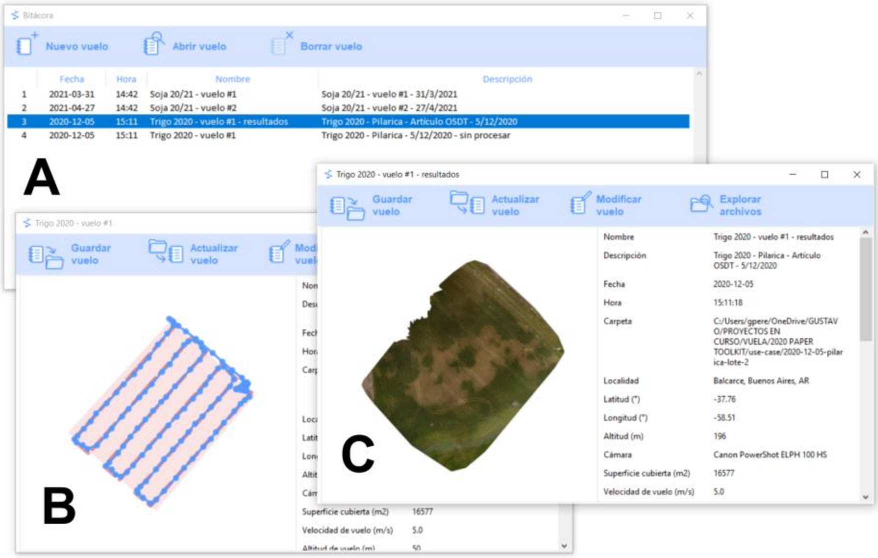

Figura 2. Captura de pantalla del software “Bitácora”. (A) Ventana principal, mostrando la lista de vuelos abiertos previamente. (B) Ventanas correspondientes a algunos de estos vuelos mostrando los datos de cada uno de ellos.

La documentación del conjunto de herramientas se compone de: 1) una "Guía de construcción" que incluye guías paso a paso para construir el dron OVLI, instalar y configurar los componentes de hardware e instalar el software, y 2) una "Guía de uso", con instrucciones para volar el dron, programar una misión autónoma, programar la cámara y procesar las imágenes. Ambos están disponibles como documentos con licencia abierta (usando una licencia Creative Commons que permite a los usuarios redistribuir y modificar el material citando la fuente), listos para descargar en formato PDF y HTML, y también como documentos “vivos” (en Google Docs) abiertos a sugerencias. Las guías tienen un diseño simple que permite la traducción automática en muchos idiomas, las cuales están disponibles en el sitio web del proyecto.

Ejemplo de uso

Se presenta aquí un caso en el que se utilizó el OSDT para obtener datos sobre la variabilidad espacial en la madurez de un cultivo de trigo, evaluada a través de un índice de vegetación que cuantifica el "verdor" del cultivo. Se relevó un área de 6000 m^2 en un lote de trigo sembrado a finales de julio de 2020 en Balcarce, Provincia de Buenos Aires, Argentina. Este lote en particular fue seleccionado por tener una madurez desigual del cultivo debido a la variabilidad

en la profundidad del suelo. Siguiendo los pasos de la "Guía de uso", primero se delimitó el área de interés (Figura 3A), se diseñó un plan de vuelo (Figura 3B) y se voló el dron OVLI el 5 de diciembre de 2020, cuando el cultivo se encontraba en la etapa de llenado de granos (Z7.7 en la escala Zadoks). Se capturaron un total de 151 imágenes a una altitud de vuelo de 50 m (Figura 3C), de las cuales 104 se utilizaron (descartando las capturadas durante el despegue y el aterrizaje) para obtener un ortomosaico con una resolución de 2 cm/pixel (Figura 3D) .

Los índices de vegetación son transformaciones de los datos obtenidos de sensores ópticos que se basan generalmente en el aumento de la reflectancia de la planta en las longitudes de onda verde y/o infrarroja, y que pueden usarse para cuantificar las variaciones espaciales y temporales en las características de la vegetación (Bannari et al., 1995). A partir de los canales rojo, verde y azul del ortomosaico se calculó un índice de vegetación (Visible Atmospherically Resistant Index, VARI) que ha sido utilizado previamente para estimar el crecimiento y la fenología del trigo (Gitelson et al., 2002; Zhou et al., 2020), con el fin de cuantificar el "verdor" del cultivo como indicador del grado de madurez (Figura 3E). Estos datos obtenidos de las imágenes del dron se compararon con el Índice de Vegetación de Diferencia Normalizada (Normalized Difference Vegetation Index, NDVI), que es el índice de vegetación más ampliamente utilizado (Jiang et al., 2006), obtenido de dos fuentes: un sensor portátil y datos satelitales.

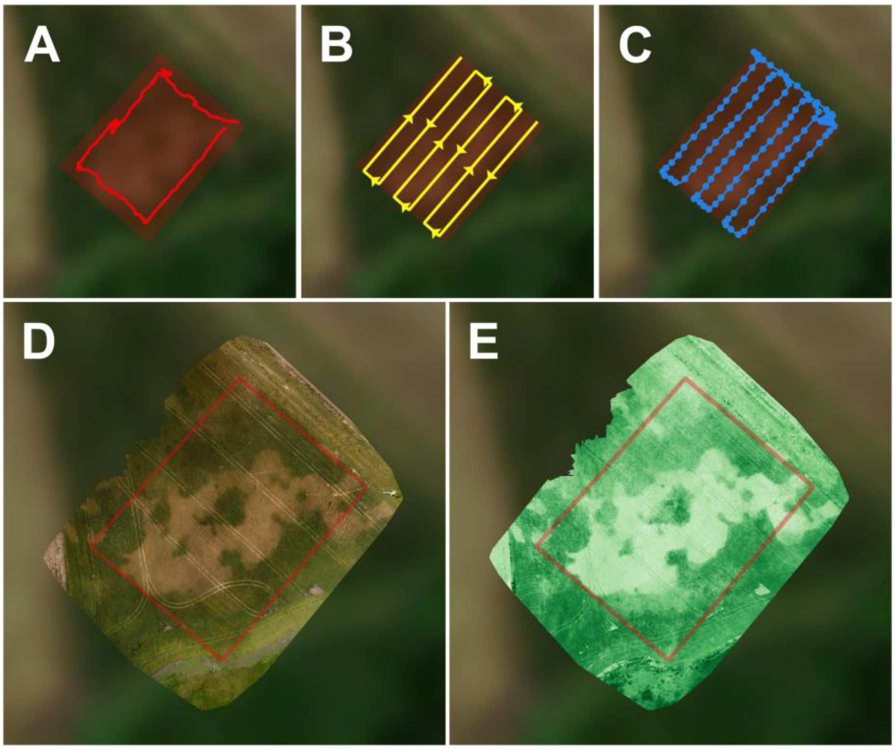

Figura 3. Pasos de captura y análisis de los datos aéreos que se siguieron en el ejemplo de uso presentado. A) identificación del área de estudio, que se realizó primero *in situ* con una aplicación de registro de ubicación (línea roja brillante) y luego se refinó dibujando manualmente un polígono rectangular (área roja semitransparente); B) diseño del plan de vuelo para cubrir el área de estudio con un patrón de tipo "grilla" o "máquina de cortar césped"; C) vuelo y captura de imágenes, representada a través de la trayectoria de vuelo real del dron (línea azul) y la posición de cada imagen capturada (círculos azules); D) ortomosaico obtenido al unir las imágenes capturadas; y E) índice de vegetación (VARI) calculado a partir de los datos del ortomosaico. La imagen de fondo es una imagen satelital de resolución 10x10m capturada 1 día después del vuelo del dron (obtenida del sitio web Sentinel Hub EO Browser bajo licencia CC-BY 4.0; Sinergise Laboratory, 2022).

Los datos satelitales de la constelación Sentinel 2 (Agencia Espacial Europea) se obtivieron del sitio web Sentinel Hub EO Browser bajo licencia CC-BY 4.0 (Sinergise Laboratory, 2022). Se utilizaron datos de las bandas B04 (rojo, longitud de onda central = 665 nm) y B08 (infrarrojo cercano, longitud de onda central = 842 nm) para el 6 de diciembre de 2020 (1 día después de la captura de imágenes mediante el dron) a una resolución de 10 m/píxel para calcular los valores NDVI para el área relevada (Figura 4A). Para la comparación entre los datos de satélite (NDVI) y dron (VARI) se delimitaron en el ortomosaico procesado 140 áreas de 10 x 10 m equivalentes a los píxeles de la imagen de satélite (Figura 4C), y se calculó el valor promedio de VARI en cada una de ellas. Algo a tener en cuenta en esta comparación es que la georreferenciación del ortomosaico estuvo basada en los datos del sensor GNSS del dron, el cual tiene usualmente una exactitud de aproximadamente 2 metros (Guo et al., 2021), por lo que la correspondencia entre estas áreas de 10 x 10 m y los píxeles de la imagen satelital podría no ser completa.

El 28 de noviembre de 2020 (7 días antes del vuelo del dron) se utilizó un sensor portátil (Greenseeker, N-tech Industries, EE. UU.) para medir el NDVI en 6 transectas paralelas a los surcos del cultivo, cada una de ellas de 70 metros de largo, y separadas entre sí 18 metros (Figura 4D). Para facilitar la correspondencia entre los datos del sensor y del dron, se colocó una marca visible al inicio de la primera transecta, y cada una de las transectas restantes se inició midiendo con cinta métrica a partir de esta marca. Las mediciones se realizaron con el sensor a 1 metro por sobre el tope del canopeo, lo cual resulta en un ancho de medición de 60 cm. Se registraron alrededor de 700 datos puntuales en cada transecta, equivalente a alrededor de 1 dato cada 10 cm. Para la comparación entre los datos del sensor NDVI portátil y los datos del dron (VARI), se promediaron los datos NDVI cada 2 metros (lo que arrojó 35 puntos de datos por transecta y 210 en total). Asimismo, en el ortomosaico procesado se delimitaron 35 áreas de 2x2m a lo largo de cada transecta, y en cada una de ellas se calculó el valor promedio de VARI.

Los datos VARI obtenidos del ortomosaico del dron se compararon con los datos NDVI de la imagen satelital y el sensor portátil, y se ajustó una ecuación exponencial de la forma $y = ax^b - c$ para cada conjunto de datos. El índice NDVI generalmente tiene una relación curvilínea con el índice de área foliar verde, mientras que el índice VARI tiene una relación más bien lineal (Gitelson et al., 2002), por lo que se puede esperar una relación curvilínea entre NDVI y VARI. Se encontró una alta correlación en ambos casos, con valores de R^2 de 0,84 y 0,88 para la comparación con los datos del satélite y del sensor de mano, respectivamente (Fig. 4E-F). También se pudo ajustar una única curva a ambos conjuntos de datos, con un R^2 de 0,86 (datos no presentados).

Discusión

En este trabajo se presentan los resultados del desarrollo de un conjunto de herramientas completo, realizado con el objeto de demostrar la posibilidad de capturar datos aéreos aptos para su uso en investigación (y también usos comerciales, *e.g.*, en agricultura) utilizando software y hardware de código abierto. El ejemplo de uso presentado demuestra su utilidad para el abordaje de múltiples preguntas de investigación. Sin embargo, la amplia gama de posibles aplicaciones de las imágenes aéreas en investigación no puede cubrirse por completo con un sólo conjunto de herramientas. La naturaleza abierta del OSDT permite, sin embargo, que sus componentes se utilicen por separado o se reemplacen por herramientas alternativas, sean éstas de código abierto o propietarias, según sea necesario. Por ejemplo, si el área de interés es significativamente más grande que la que se relevó en el ejemplo presentado, el tiempo de vuelo del dron OVLI no sería suficiente. En ese caso, un dron de tipo “ala fija”, como el dron de código abierto “Asa-Branca-I” (Mesquita et al., 2021), podría brindar la capacidad de cubrir más de 100 ha en un solo vuelo (aunque con menor resolución de imagen). Y si el costo de un dron es una limitante, o si los drones no se pueden usar

debido a regulaciones locales, también se pueden capturar imágenes aéreas usando barriletes o cometas (Anderson et al., 2016). También es posible utilizar las herramientas de software de procesamiento, análisis y gestión de imágenes del kit de herramientas con imágenes aéreas obtenidas con drones propietarios.

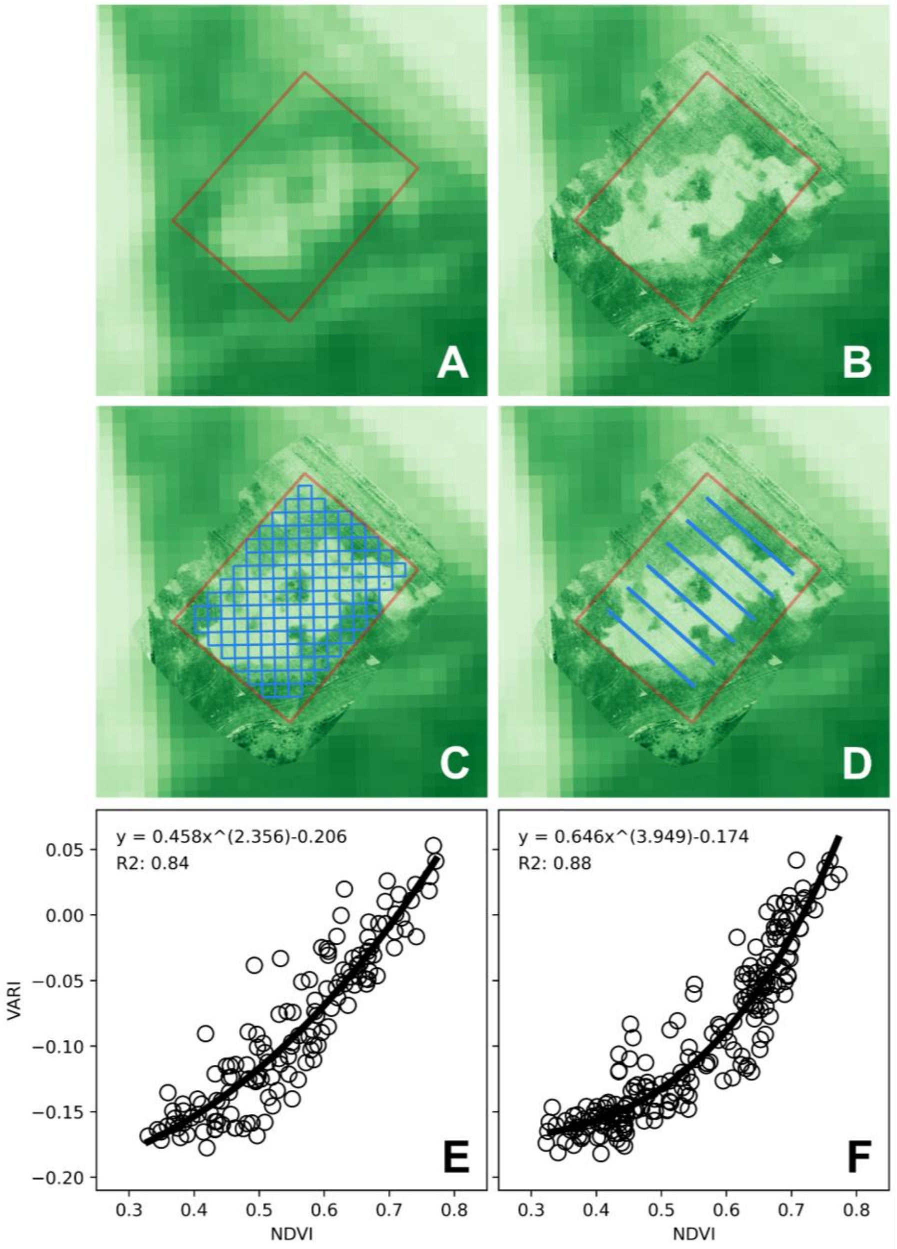

Figura 4. Comparación entre los datos obtenidos mediante el OSDT y los datos obtenidos de una imagen satelital o de un sensor manual. A) Imagen satelital NDVI de Sentinel 2; B) Imagen VARI del ortomosaico obtenido mediante el dron; C) Imagen del dron VARI que muestra las áreas de 10x10m equivalentes a los píxeles de la imagen satelital delimitados en el ortomosaico procesado; D) transectas utilizados para la medición con sensor manual; E) relación entre NDVI de la imagen satelital y VARI del dron; y F) relación entre NDVI del sensor portátil y VARI del dron. La imagen satelital fue obtenida del sitio web Sentinel Hub EO Browser bajo licencia CC-BY 4.0 (Sinergise Laboratory, 2022)

Las dudas sobre la calidad y la confiabilidad de las herramientas de código abierto (especialmente el hardware) a veces pueden limitar su uso (Parker et al., 2021). En este trabajo intentamos superar esta limitación comparando los resultados obtenidos con el OSDT con datos de imágenes satelitales y de un sensor portátil comercial, encontrando una alta correlación para ambos instrumentos. Los dos sensores tomados como referencia en las comparaciones se encuentran entre los más ampliamente utilizados por los agricultores para el monitoreo de cultivos en los últimos 20 años (Mulla y Khosla, 2016), ambos hacen uso del índice de vegetación más utilizado (NDVI; Jiang et al., 2006), por lo que pueden ser considerados como una referencia confiable. Los estudios con cámaras RGB disponibles en el mercado, similares a la que se usa en el OSDT, han demostrado que pueden producir mediciones robustas de índices de vegetación (Svensgaard et al., 2019), que pueden mejorarse aún más mediante calibraciones radiométricas (Svensgaard et al., 2021), obteniendo resultados comparables a los de las cámaras multiespectrales que pueden costar varias veces más (Holman et al., 2019; Ashapure et al., 2019; Costa et al., 2020; Davidson et al., 2022), y con una mayor resolución espacial (Herzig et al., 2021). Aunque no se muestra en este trabajo, el OSDT también se puede utilizar para generar mapas de elevación digitales y nubes de puntos 3D del terreno estudiado. Los estudios que utilizaron herramientas similares demostraron que es posible obtener resultados comparables a los de los costosos sistemas LiDAR (Light Detection And Ranging), cuando se utilizan puntos de control en tierra apropiados (Zahawi et al., 2015; Zhang et al., 2021).

También suelen plantearse dudas acerca de la sostenibilidad de las herramientas de código abierto (Carver et al., 2022), especialmente cuando éstas no cuentan con un modelo de negocio que las financie, lo cual es importante dado que la disponibilidad a largo plazo de las herramientas es necesaria para la reproducibilidad y replicabilidad (Hocquet y Weiber, 2021). A pesar de esto, se suele considerar que el software utilizado en investigación debe ser de código abierto, ya que esto posibilita la validación, reutilización y mejora a lo largo del tiempo (Jiménez et al., 2017; Antz et al., 2021), mientras que, por el contratio, el software propietario suele ser un obstáculo para la reproducibilidad (Konkol et al., 2019). La sostenibilidad del hardware de código abierto no ha sido tan extensamente estudiada (Li y Seering, 2019), pero se suele considerar que otorga una mayor seguridad a largo plazo a los proyectos de investigación que lo utilizan, debido a la posibilidad de reparación en caso que el proveedor original deje el mercado (Chagas, 2018). Proponemos, por lo tanto, que utilizar el OSDT para la captura de datos aéreos para investigación puede considerarse como una opción más sostenible que los sistemas comerciales de código cerrado.

Si bien ha habido mejoras técnicas significativas en drones y sensores en los últimos años, se ha prestado poca atención a la gestión y almacenamiento de conjuntos de datos cada vez más grandes y complejos que son el resultado de las operaciones con drones (Wyngaard et al., 2019). Hay pocos estándares para la gestión, el intercambio o la publicación de datos de drones, lo que dificulta la colaboración y la reproducibilidad (Thomer et al., 2021). Los paquetes de drones comerciales suelen ofrecer soluciones completas, pero generalmente a cambio de costosas licencias, menor interoperabilidad con otras herramientas, o menor posibilidad de personalización. Las herramientas de código abierto ofrecen un ecosistema más flexible pero fragmentado, y los usuarios pueden verse intimidados por la necesidad de lidiar con muchos componentes individuales. En el OSDT se desarrolló el software "Bitácora" con el objetivo de ayudar a superar este problema, al brindar una forma de centralizar todos los archivos y datos que se generan en las diferentes etapas del proceso de captura de datos aéreos, que de otra manera tienen que ser gestionados y visualizados usando muchas herramientas diferentes. Al tratarse de un software de código abierto, es posible ampliarlo para incorporar otros formatos de datos y tipos de archivos (por ejemplo, planes de vuelo para drones propietarios, imágenes de cámaras multiespectrales, etc.). También ayuda al usuario a recopilar todos los datos generados en una sola carpeta, agregando los metadatos correspondientes, lo que ayuda a compartir abiertamente los datos de investigación en formatos reutilizables e interoperables. "Bitácora" puede extraer

automáticamente parte de los metadatos requeridos en el Marco Mínimo de Información (“Minimum Information Framework”) para usuarios de drones (propuesto por Thomer et al., 2021), ayudando así a los usuarios a que sus datos sean “FAIR” (localizables, accesibles, interoperables y reutilizables, por sus siglas en inglés).Otro software de código abierto con un objetivo similar es “DroneDB” (DroneDB Authors, 2020), que ayuda especialmente a compartir conjuntos de datos de imágenes, ortomosaicos y otros productos de drones a través de una interfaz en la nube, pero sin proporcionar metadatos de vuelo. Por lo tanto, “Bitácora” es un componente menor pero clave del OSDT, ya que ayuda a amalgamar los componentes del conjunto de herramientas y a utilizarlo para promover los objetivos de la ciencia abierta.

Conclusión

En este documento se presenta el “Open Science Drone Toolkit”, que permite al usuario realizar todas las tareas necesarias para obtener datos a partir de imágenes aéreas. Los datos obtenidos con este conjunto de herramientas sobre un campo de trigo fueron comparados con datos de imágenes satelitales y un sensor portátil comercial, y se encontró una alta correlación para ambos instrumentos. Estos resultados demuestran la posibilidad de capturar datos aéreos aptos para su uso en investigación utilizando software y hardware de código abierto, asequibles, accesibles y personalizables, y protocolos de trabajo abiertos.

Agradecimientos

Queremos agradecer las contribuciones de los participantes de los talleres del proyecto Vuela y los colaboradores en línea que hicieron posible la construcción de este kit de herramientas, especialmente Loulou Jude, Daniela Muñoz, Lot Amorós, Guillermo Pereyra Irujo, Nicolás Narváez, Carla Alvial, Fernando Yamada, John Arancibia , Constanza Alberio, Vicente Dimuro y Stevens Azima. Queremos agradecer a la Junta de Vecinos Teniente Merino Alto, Junta de Vecinos Francisco Werchez, Universidad Católica, INIA Rayentué (Chile), INTA Balcarce, Club Social de Innovación Balcarce, R'lyeh Hacklab, INTA Marcos Juárez y Universidad Nacional de Cuyo (Argentina), IPTA Capitán Miranda (Paraguay), Universidade Federal do Rio Grande do Sul (Brasil) e INIA La Estanzuela (Uruguay) por proporcionar los lugares para los talleres. También queremos agradecer a Abril Pereyra Molina y Julián Pereyra Molina por su asistencia con las mediciones de campo.

Financiamiento

El financiamiento para este proyecto fue proporcionado por Mozilla Foundation (Mozilla Science Lab Mini Grant) a GPI y PB, Knowledge/Culture/Ecologies (KCE2017) a PB, Shuttleworth Foundation (Flash Grant) a PB, Programa Cooperativo para el Desarrollo Tecnológico Agroalimentario y Agroindustrial del Cono Sur (PROCISUR) a GPI, Instituto Nacional de Tecnología Agropecuaria (PNCYO-1124072 y 2019-PD-E3-I060) a GPI. Los patrocinadores no tuvieron ningún papel en el diseño del estudio, la recopilación y el análisis de datos, la decisión de publicar o la preparación del manuscrito.

Disponibilidad de los datos

Los detalles del "Open Science Drone Toolkit" se pueden encontrar en https://vuela.cc/, y los datos del ejemplo de uso están disponibles en https://osf.io/t7y6x/.

Referencias

Amorós L, Varona N, Boronat R, Rangholia C. Flone: una plataforma para que los smartphones puedan volar. 2015. Disponible en: https://flone.cc/

Anderson K, Griffiths D, DeBell L, Hancock S, Duffy JP, Shutler JD, et al. A Grassroots Remote Sensing Toolkit Using Live Coding, Smartphones, Kites and Lightweight Drones. PLOS ONE. 2016;11(5):e0151564.

Anzt H, Bach F, Druskat S, Löffler F, Loewe A, Renard BY, et al. An environment for sustainable research software in Germany and beyond: current state, open challenges, and call for action. F1000Research. 2021;9:295.

Arancio J, Dosemagen S. Bringing Open Source to the Global Lab Bench. Issues in Science and Technology. 2022. Disponible en: https://issues.org/open-source-science-hardware-gosh-arancio-dosemagen/

ArduPilot Project. ArduPilot. ArduPilot.org. 2016. Disponible en: https://ardupilot.org

Ashapure A, Jung J, Chang A, Oh S, Maeda M, Landivar J. A Comparative Study of RGB and Multispectral Sensor-Based Cotton Canopy Cover Modelling Using Multi-Temporal UAS Data. Remote Sensing. 2019;11(23):2757.

Assmann JJ, Kerby JT, Cunliffe AM, Myers-Smith IH. Vegetation monitoring using multispectral sensors — best practices and lessons learned from high latitudes. J Unmanned Veh Syst. 2019;7(1):54–75.

Bahlai C, Bartlett L, Burgio K, Fournier A, Keiser C, Poisot T, et al. Open Science Isn't Always Open to All Scientists. Am Sci. 2019;107(2):78.

Bannari A, Morin D, Bonn F, Huete AR. A review of vegetation indices. Remote Sens Rev. 1995;13(1–2):95–120.

Bernaldo P, Pereyra Irujo G. Proyecto "Vuela." Liinc Em Rev. 2018;14(1)

Boulton G, Campbell P, Collins B, Elias P, Hall W, Laurie G, et al. Science as an open enterprise. London: The Royal Society; 2012.

Boulton G, Rawlins M, Vallance P, Walport M. Science as a public enterprise: the case for open data. The Lancet. 2011;377(9778):1633–5.

Carver JC, Weber N, Ram K, Gesing S, Katz DS. A survey of the state of the practice for research software in the United States. PeerJ Comput Sci. 2022;8:e963.

Chabot D. Trends in drone research and applications as the Journal of Unmanned Vehicle Systems turns five. J Unmanned Veh Syst. 2018;6(1):vi–xv.

Chagas AM. Haves and have nots must find a better way: The case for open scientific hardware. PLOS Biol. 2018;16(9):e3000014.

CHDK Development Team. Canon Hack Development Kit. 2007. Disponible en: https://chdk.fandom.com/wiki/CHDK

Costa L, Nunes L, Ampatzidis Y. A new visible band index (vNDVI) for estimating NDVI values on RGB images utilizing genetic algorithms. Computers and Electronics in Agriculture. 2020;172:105334.

Davidson C, Jaganathan V, Sivakumar AN, Czarnecki JMP, Chowdhary G. NDVI/NDRE prediction from standard RGB aerial imagery using deep learning. Computers and Electronics in Agriculture. 2022;203:107396.

Diederich B, Müllenbroich C, Vladimirov N, Bowman R, Stirling J, Reynaud EG, et al. CAD we share? Publishing reproducible microscope hardware. Nat Methods. 2022;1–5.

DroneDB Authors. DroneDB - Effortless Aerial Data Management and Sharing. 2020. Disponible en: https://dronedb.app/

Eskandari R, Mahdianpari M, Mohammadimanesh F, Salehi B, Brisco B, Homayouni S. Meta-analysis of Unmanned Aerial Vehicle (UAV) Imagery for Agro-environmental Monitoring Using Machine Learning and Statistical Models. Remote Sens. 2020;12(21):3511.

Forero DA, Curioso WH, Patrinos GP. The importance of adherence to international standards for depositing open data in public repositories. BMC Res Notes. 2021;14(1):405.

Gitelson AA, Kaufman YJ, Stark R, Rundquist D. Novel algorithms for remote estimation of vegetation fraction. Remote Sens Environ. 2002;80(1):76–87.

Guo W, Carroll ME, Singh A, Swetnam TL, Merchant N, Sarkar S, et al. UAS-Based Plant Phenotyping for Research and Breeding Applications. Plant Phenomics. 2021;2021:9840192.

Hanrahan BV, Maitland C, Brown T, Chen A, Kagame F, Birir B. Agency and Extraction in Emerging Industrial Drone Applications: Imaginaries of Rwandan Farm Workers and Community Members. Proc ACM Hum-Comput Interact. 2021;4(CSCW3):233:1-233:21.

Herzig P, Borrmann P, Knauer U, Klück HC, Kilias D, Seiffert U, et al. Evaluation of RGB and Multispectral Unmanned Aerial Vehicle (UAV) Imagery for High-Throughput Phenotyping and Yield Prediction in Barley Breeding. Remote Sensing. 2021;13(14):2670.

Himmelstein DS, Romero AR, Levernier JG, Munro TA, McLaughlin SR, Greshake Tzovaras B, et al. Sci-Hub provides access to nearly all scholarly literature. eLife. 2018;7:e32822.

Hocquet A, Wieber F. Epistemic issues in computational reproducibility: software as the elephant in the room. Euro Jnl Phil Sci. 2021;11(2):38.

Holman FH, Riche AB, Castle M, Wooster MJ, Hawkesford MJ. Radiometric Calibration of 'Commercial off the Shelf' Cameras for UAV-Based High-Resolution Temporal Crop Phenotyping of Reflectance and NDVI. Remote Sens. 2019;11(14):1657.

Hull D. Openness and Secrecy in Science: Their Origins and Limitations. Sci Technol Hum Values. 1985;10(2):4–12.

Jewan SYY, Pagay V, Billa L, Tyerman SD, Gautam D, Sparkes D, et al. The feasibility of using a low-cost near-infrared, sensitive, consumer-grade digital camera mounted on a commercial UAV to assess Bambara groundnut yield. International Journal of Remote Sensing. 2022;43(2):393-423.

Jiang Z, Huete AR, Chen J, Chen Y, Li J, Yan G, et al. Analysis of NDVI and scaled difference vegetation index retrievals of vegetation fraction. Remote Sens Environ. 2006;101(3):366–78.

Jiménez RC, Kuzak M, Alhamdoosh M, Barker M, Batut B, Borg M, et al. Four simple recommendations to encourage best practices in research software. F1000Research 2017;6.

Konkol M, Kray C, Pfeiffer M. Computational reproducibility in geoscientific papers: Insights from a series of studies with geoscientists and a reproduction study. International Journal of Geographical Information Science. 2019;33(2):408-29.

Li Z, Seering W. Does Open Source Hardware Have a Sustainable Business Model? An Analysis of Value Creation and Capture Mechanisms in Open Source Hardware Companies. Proceedings of the Design Society: International Conference on Engineering Design. 2019;1(1):2239-48.

Meier L, Tanskanen P, Heng L, Lee GH, Fraundorfer F, Pollefeys M. PIXHAWK: A micro aerial vehicle design for autonomous flight using onboard computer vision. Auton Robots. 2012;33(1):21–39.

Mesquita GP, Rodríguez-Teijeiro JD, Oliveira RR de, Mulero-Pázmány M. Steps to build a DIY low-cost fixed-wing drone for biodiversity conservation. PLOS ONE. 2021;16(8):e0255559.

Mulla D, Khosla R. Historical Evolution and Recent Advances in Precision Farming. En: Lal R, Stewart BA, Eds. Soil Specific Farming: Precision Agriculture. CRS Press: Boca Raton, FL, USA, 2016.

O'Connor J, Smith MJ, James MR. Cameras and settings for aerial surveys in the geosciences: Optimising image data. Prog Phys Geogr Earth Environ. 2017;41(3):325–44.

Open Source Hardware Association. Open Source Hardware (OSHW) Statement of Principles 1.0. Open Source Hardware Association. 2016. Disponible en: https://www.oshwa.org/definition/

Open Source Initiative. The Open Source Definition. 2007. Disponible en: https://opensource.org/osd

OpenDroneMap Authors. OpenDroneMap: A command line toolkit to generate maps, point clouds, 3D models and DEMs from drone, balloon or kite images. 2020. Disponible en: https://github.com/OpenDroneMap/ODM

Paneque-Gálvez J, Vargas-Ramírez N, Napoletano BM, Cummings A. Grassroots Innovation Using Drones for Indigenous Mapping and Monitoring. Land. 2017;6(4):86.

Parker A, Dosemagen S, Molloy J, Bowser A, Novak A. Open Hardware: An Opportunity to Build Better Science. The Wilson Center; 2021. Disponible en: https://diplomacy21-adelphi.wilsoncenter.org/sites/default/files/media/uploads/documents/STIP%20Open%20Hardware%20An%20Opportunity%20to%20Build%20Better%20Science_0.pdf

Pell T, Li JYQ, Joyce KE. Demystifying the Differences between Structure-from-Motion Software Packages

for Pre-Processing Drone Data. Drones. 2022;6(1):24.

Piwowar H, Priem J, Larivière V, Alperin JP, Matthias L, Norlander B, et al. The state of OA: a large-scale analysis of the prevalence and impact of Open Access articles. PeerJ. 2018;6:e4375.

Pixhawk Project. Pixhawk: the hardware standard for open-source autopilots. 2014. Disponible en: https://pixhawk.org/

Proyecto Vuela. Proyecto Vuela: ciencia libre con drones. Proyecto Vuela. 2018. Disponible en: http://vuela.cc/

QGIS Development Team. QGIS: a Free and Open Source Geographic Information System. 2002. Disponible en: https://www.qgis.org/

Ravindran S. How DIY technologies are democratizing science. Nature. 2020;587(7834):509–11.

Rusnák M, Sládek J, Kidová A, Lehotský M. Template for high-resolution river landscape mapping using UAV technology. Measurement. 2018;115:139–51.

Sankaran S, Zhou J, Khot LR, Trapp JJ, Mndolwa E, Miklas PN. High-throughput field phenotyping in dry bean using small unmanned aerial vehicle based multispectral imagery. Computers and Electronics in Agriculture. 2018;151:84-92.

Short J, Mackay R, Robustini M. ArduCopter. 2015. Disponible en: https://github.com/ArduPilot/ardupilot/blob/master/ArduCopter/Copter.cpp

Sinergise Laboratory. EO Browser. 2022. Disponible en: https://apps.sentinel-hub.com/eo-browser/?zoom=18&lat=-37.76428&lng=-58.51554&datasetId=S2L2A&fromTime=2020-12-06&toTime=2020-12-07

Svensgaard J, Jensen SM, Christensen S, Rasmussen J. The importance of spectral correction of UAV-based phenotyping with RGB cameras. Field Crops Res. 2021;269:108177.

Svensgaard J, Jensen SM, Westergaard JC, Nielsen J, Christensen S, Rasmussen J. Can reproducible comparisons of cereal genotypes be generated in field experiments based on UAV imagery using RGB cameras? Eur J Agron. 2019;106:49–57.

Thomer A, Barbieri L, Wyngaard J, Swanz S. A Minimum Information Framework for capturing FAIR data with small Uncrewed Aircraft Systems. 2021; Disponible en: https://eartharxiv.org/repository/view/2593/

Tmušić G, Manfreda S, Aasen H, James MR, Gonçalves G, Ben-Dor E, et al. Current Practices in UAS-based Environmental Monitoring. Remote Sens. 2020;12(6):1001.

UNESCO. UNESCO Recommendation on Open Science. UNESCO; 2021. Disponible en: https://unesdoc.unesco.org/ark:/48223/pf0000379949.locale=en

Vargas-Ramírez N, Paneque-Gálvez J. The Global Emergence of Community Drones (2012–2017). Drones. 2019;3(4):76.

Vautherin J, Rutishauser S, Schneider-Zapp K, Choi HF, Chovancova V, Glass A, et al. Photogrammetric accuracy and modeling of rolling shutter cameras. En: ISPRS Annals of the Photogrammetry, Remote Sensing and Spatial Information Sciences. Copernicus GmbH; 2016. P. 139–46. Disponible en: https://www.isprs-ann-photogramm-remote-sens-spatial-inf-sci.net/III-3/139/2016/

Walters WP. Code Sharing in the Open Science Era. J Chem Inf Model. 2020;60(10):4417–20.

Wolff B, Schlagwein D. From Open Science to Open Source (and beyond): A Historical Perspective on Open Practices without and with IT. En: 17th International Symposium on Open Collaboration. New York, NY, USA: Association for Computing Machinery; 2021. P. 1–11. (OpenSym 2021). doi: 10.1145/3479986.3479990

Wyngaard J, Barbieri L, Thomer A, Adams J, Sullivan D, Crosby C, et al. Emergent Challenges for Science sUAS Data Management: Fairness through Community Engagement and Best Practices Development. Remote Sens. 2019;11(15):1797.

Zahawi RA, Dandois JP, Holl KD, Nadwodny D, Reid JL, Ellis EC. Using lightweight unmanned aerial vehicles to monitor tropical forest recovery. Biol Conserv. 2015;186:287–95.

Zhang F, Hassanzadeh A, Kikkert J, Pethybridge SJ, van Aardt J. Comparison of UAS-Based Structure-from-Motion and LiDAR for Structural Characterization of Short Broadacre Crops. Remote Sens. 2021;13(19):3975.

Zhou M, Ma X, Wang K, Cheng T, Tian Y, Wang J, et al. Detection of phenology using an improved shape model on time-series vegetation index in wheat. Comput Electron Agric. 2020;173:105398.
