# Supplementary material for: Open Science Drone Toolkit: Open source hardware and software for aerial data capture
Source: PLoS One. 2023 Apr 6;18(4):e0284184. doi: 10.1371/journal.pone.0284184 (PMC10079087; doi:10.1371/journal.pone.0284184)
Supplement: Attachment — Submitted filename: response-to-reviewers-rev2.pdf [file pone.0284184.s004.pdf]

Review Comments to the Author - Reviewer #1

Thank you for the opportunity to review this new version of the manuscript. You have carefully addressed the reviewer comments. I am pleased to recommend publication with the following minor recommendation:

I still get a sense that the comparative data are being presented as "reference" and as an "evaluation" rather than simply a comparison; the above words probably require a different approach, which I don't believe is necessary; I suggest changing these words to "comparison" or to a close synonym.

We appreciate the Reviewer's recommendation for publication. As suggested, the words "reference" and "evaluation" were deleted or replaced with "comparison" or similar words, as follows:

Lines 28-30 (Abstract): "Performance was ***evaluated*** by…" was replaced by "Data obtained with this toolkit … was ***compared*** to…", and "both ***reference*** instruments" was changed to simply "both instruments".

Line 188: "two ***reference*** sources" was changed to "two sources".

Line 248: "both ***reference*** instruments" was replaced by "both instruments".

Lines 288-290: Same changes as in the Abstract

Journal requirements

Please review your reference list to ensure that it is complete and correct. If you have cited papers that have been retracted, please include the rationale for doing so in the manuscript text, or remove these references and replace them with relevant current references. Any changes to the reference list should be mentioned in the rebuttal letter that accompanies your revised manuscript. If you need to cite a retracted article, indicate the article's retracted status in the References list and also include a citation and full reference for the retraction notice.

The reference list was double-checked, and we believe it is complete and correct. We also checked that none of the cited papers had been retracted.
